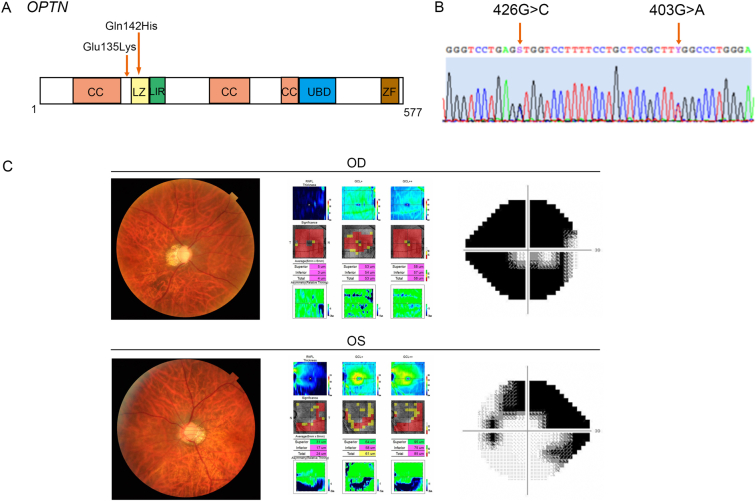# Identification of *OPTN* p.(Asn51Thr): A novel pathogenic variant in primary open-angle glaucoma

**DOI:** 10.1016/j.gimo.2023.100839

**Published:** 2023-10-31

**Authors:** Yukihiro Shiga, Kazuki Hashimoto, Kosuke Fujita, Shigeto Maekawa, Kota Sato, Shintaroh Kubo, Kazuhide Kawase, Kana Tokumo, Yoshiaki Kiuchi, Sotaro Mori, Makoto Nakamura, Takeshi Iwata, Koji M. Nishiguchi, Toru Nakazawa

**Affiliations:** 1 Department of Ophthalmology, Tohoku University Graduate School of Medicine, Sendai, Miyagi, Japan; 2 Neuroscience Division, Centre de Recherche du Centre Hospitalier de l'Université de Montréal, Montréal, Québec, Canada; 3 Department of Neuroscience, Université de Montréal, Montréal, Québec, Canada; 4 Department of Ophthalmology, Nagoya University Graduate School of Medicine, Nagoya, Aichi, Japan; 5 Department of Anatomy and Cell Biology, McGill University, Montréal, Québec, Canada; 6 Department of Biological Science, Grad. Sch. of Sci, The University of Tokyo, Tokyo, Japan; 7 Yasuma Eye Clinic, Nagoya, Aichi, Japan; 8 Department of Ophthalmology Protective Care for Sensory Disorders, Nagoya University Graduate School of Medicine, Nagoya, Aichi, Japan; 9 Department of Ophthalmology and Visual Science, Graduate School of Biomedical and Health Sciences, Hiroshima University, Hiroshima, Japan; 10 Division of Ophthalmology, Department of Surgery, Kobe University Graduate School of Medicine, Kobe, Japan; 11 Division of Molecular and Cellular Biology, National Institute of Sensory Organs, National Hospital Organization Tokyo Medical Center, Tokyo, Japan; 12 Department of Retinal Disease Control, Tohoku University Graduate School of Medicine, Sendai, Miyagi, Japan; 13 Department of Advanced Ophthalmic Medicine, Tohoku University Graduate School of Medicine, Sendai, Miyagi, Japan; 14 Department of Ophthalmic Imaging and Information Analytics, Tohoku University Graduate School of Medicine, Sendai, Miyagi, Japan

**Keywords:** Genetics, Glaucoma, *Myocilin* (*MYOC*), *Optineurin* (*OPTN*), *TANK Binding Kinase 1* (*TBK1*)

## Abstract

**Purpose:**

Pathogenic variants in *TBK1*, *MYOC*, and *OPTN* are associated with primary open-angle glaucoma (POAG) with severe visual field defects. This study aims to understand further POAG-related pathogenic variant(s) based on a cohort of East Asian populations that have not been well-characterized.

**Methods:**

We conducted a comprehensive screening of *TBK1*, *MYOC*, and *OPTN* variants in 174 POAG Japanese patients, followed by 8380 population-specific genome sequencing data references, segregation analysis, and functional protein assays to determine pathogenic variants.

**Results:**

Despite the small sample size, 4 variants were novel, 2 of which p.(Cys5Trp) and p.(Thr293Met) were in the *MYOC* gene, and 2 p.(Asn51Thr), and p.(Gln142His) were in the *OPTN*. Notably, the *OPTN* p.(Asn51Thr) missense variant adjacent to the p.(Glu50Lys) variant, a well-known POAG pathogenic variant, was segregated from all proband’s family members with POAG. Moreover, in silico and in vitro analyses revealed that the *OPTN* p.(Asn51Thr) protein increased binding instability, interactions of the OPTN-TBK1 complex, and enhanced protein insolubility, likewise the p.(Glu50Lys) protein.

**Conclusion:**

Our findings may provide further genetic insights into rare variants of POAG and support the clear conclusion that *OPTN* p.(Asn51Thr) is a novel likely pathogenic variant.

## Graphical abstract

**Figure:**
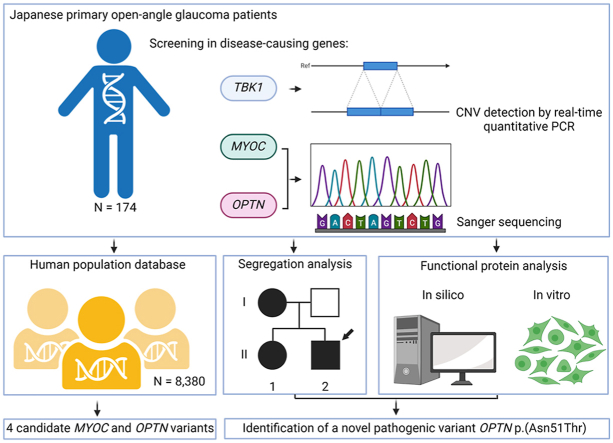

## Introduction

The hallmark of glaucoma, the leading cause of irreversible blindness, is a progressive loss of retinal ganglion cells (RGCs) and their axons that correspond to patterns of spatial visual field defects, affecting 111.8 million people in 2040 globally.^1^^,^^2^ Primary open-angle glaucoma (POAG), the most common type of the disease, is considered an age-related and multifactorial disease, and elevated intraocular pressure (IOP) is the only significant treatable risk factor for disease development and progression.^3^ However, POAG can also occur when IOP is in the normal range, a condition known as normal-tension glaucoma (NTG).^4^ Previous population surveys have shown that the majority of POAG cases in East Asians are NTG, notably in the Tajimi Study in Japan, where NTG accounted for 92% of POAG cases.^5^ In contrast, European and African ancestries have a smaller proportion of NTG, ranging from 21% to 39%.^6^, ^7^, ^8^ Although the pathogenesis of NTG is not completely understood, these regional and ethnic differences in the prevalence of NTG suggest that genetic predisposition may contribute substantially to the phenotypic heterogeneity of the disease.

Indeed, family history is a well-established risk factor for POAG. First-degree relatives of patients are 3 to 9 times more likely to develop the disease than the general population,^9^^,^^10^ supporting the highly heritable aspect of POAG. A recent genome-wide meta-analysis has successfully identified 127 susceptible common variants of POAG, with most loci showing broadly consistent effects across European, Asian, and African ancestries.^11^ Furthermore, a glaucoma polygenic risk score combining POAG-associated common and rare variants was shown to predict glaucoma progression and surgical intervention in advanced disease, allowing the development of personalized approaches for early therapeutic intervention for high-risk individuals.^12^ Nevertheless, the majority of studies on rare variants of POAG have been conducted in cohorts of European ancestries, and understanding the involvement of POAG rare variants in East Asians remains largely unknown.

At least 5% of adult-onset POAG cases are caused primarily by rare variants in 1 of 3 genes: *MYOC* [HGNC:7610], *OPTN* [HGNC:17142], or *TBK1* [HGNC:11584]^13^ Myocilin is expressed mainly in the trabecular meshwork, and more than 90% of rare variants in *MYOC* previously associated with POAG are located in the olfactomedin domain.^14^ POAG cases with *MYOC* variants are typically associated with increased IOP. However, recent studies demonstrated that *MYOC* p.(Gln368Ter), which is thought to be the most common *MYOC*-related variant in European populations, is also found in patients with NTG,^15^ indicating that *MYOC* variants are associated with glaucoma that occurs within a broader range of IOP. In contrast, optineurin and TBK1 proteins are expressed in the retina, particularly in RGCs, and they are known to interact.^16^ The missense variant of *OPTN* p.(Glu50Lys)^17^ and *TBK1* DNA copy-number variations (CNVs)^18^ cause early-onset familial NTG with autosomal dominant inheritance. The *OPTN* p.(Glu50Lys) protein enhances TBK1-OPTN binding and increases protein insolubility, promoting selective RGCs death and reactive gliosis.^19^ Thus, further identifying rare variants in these POAG-related genes in East Asians and their pathogenicity is essential to establish precision medicine to prevent blindness due to glaucoma.

In this study, we conducted a comprehensive *TBK1* DNA CNVs, *MYOC*, and *OPTN* variants screening in 174 Japanese POAG patients with a strong genetic predisposition. Using a Japanese-specific reference panel of 8380 individuals, we identified 4 rare variants within *MYOC* and 3 variants within *OPTN*. Strikingly, a novel *OPTN* p.(Asn51Thr) missense variant adjacent to the p.(Glu50Lys) variant, a known pathogenic variant in NTG, was detected in a patient with NTG. In addition, the *OPTN* p.(Asn51Thr) variant was isolated from proband’s family members diagnosed with glaucoma. Moreover, in silico and in vitro analyses revealed that the *OPTN* p.(Asn51Thr) protein increased the binding instability of the OPTN-TBK1 complex and enhanced protein insolubility; similar to the p.(Glu50Lys) protein. Our findings shed new insights into rare variants of POAG in East Asians and support the clear conclusion that *OPTN* p.(Asn51Thr) is a likely pathogenic variant.

## Materials and Methods

### Subjects

An overview of the experimental design, consisting of 4 steps of analysis, is shown in Figure 1.

**Figure 1:**
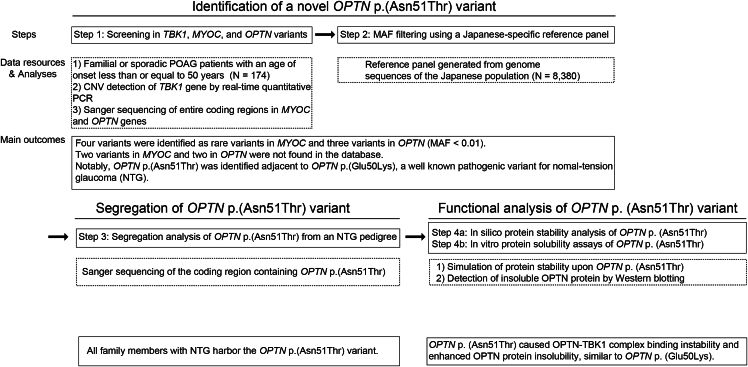
**Study design.** This figure summarizes the 4 steps of this study and the data resources and primary analyses/outcomes for each step. CNVs, copy-number variations; MAF, minor allele frequency; NTG, normal-tension glaucoma; PCR, polymerase chain reaction; POAG, primary open-angle glaucoma.

One hundred seventy-four patients with POAG who met the criteria with (1) a family history of glaucoma in the second-degree relative or (2) an age of onset less than or equal to 50 years were enrolled in this study. These strict criteria allowed the selection of glaucoma patients with a strong genetic predisposition. The POAG patients were recruited at the Institutes related to Tohoku University or the Japan Glaucoma Society Omics Group.

All POAG subjects were diagnosed by glaucoma specialists and fulfilled the following diagnostic criteria: (1) glaucomatous optic disc changes, including neuroretinal rim thinning, notching, or cupping; (2) glaucomatous visual field defect measured by Humphrey automated or Goldmann perimetry; (3) normal open-angle measured by gonioscopy; and (4) no history of secondary or angle-closure glaucoma.

Based on IOP values measured with Goldmann applanation tonometry at the time of untreated or diagnosis, the cases were further classified into 44 high-tension glaucoma, defined as peak IOP greater than or equal to 22 mmHg, and 117 NTG, defined as peak IOP less than 22 mmHg. In 13 cases, peak IOP data were unavailable and classified as unknown. The demographic characteristics of the study subjects are shown in Supplemental Table 1.

All subjects were enrolled in the study after written informed consent and approval from the Institutional Review Board following the Declaration of Helsinki.

### Polymerase chain reaction–based detection of DNA CNVs in *TBK1*

To assess *TBK1* DNA CNVs, we initially conducted TaqMan Copy-Number Assays with a specific primer for *TBK1* (Hs06980763_cn) and its reference probe (TaqMan Copy-Number Reference Assay) as a screening. Genomic DNA was extracted from peripheral blood or saliva and purified with the QIAsymphony DNA Mini Kit or the Qiagen QIAamp Blood Kit (Qiagen). All extracted DNA samples were triplicates, and polymerase chain reaction (PCR) amplification was performed using a 7500 Fast Real-Time PCR system (Applied Biosystems, Foster City) according to the manufacturer’s protocol. The copy number of *TBK1* was analyzed from real-time PCR products using the CopyCaller Software.

Digital droplet PCR (ddPCR) was subsequently conducted to confirm *TBK1* duplication. For the ddPCR, genomic DNA from the glaucoma patients was amplified with predesigned *TBK1* primer (Bio-Rad) and analyzed with a QX200 Droplet Generator and a QX200 Droplet Reader (Bio-Rad) as manufactural protocol. In both TaqMan Copy-Number Assays and ddPCR protocols, a case with previously confirmed *TBK1* gene duplication^20^ was used as a positive control and 3 healthy individuals as negative controls.

### Sanger sequencing of *MYOC* and *OPTN* in the protein-coding regions

Sanger sequencing was used to sequence the coding region of *MYOC* and *OPTN*. All 3 exons encoding a 504 amino acid protein within the *MYOC* and 13 exons encoding a 577 amino acid protein within the *OPTN* were amplified by PCR. Primers used for nucleic acid amplification were designed using the Primer-BLAST (https://www.ncbi.nlm.nih.gov/tools/primer-blast/index.cgi). The sequences of these primers are shown in Supplemental Table 2. PCR fragments were purified by ExoSAP-IT (USB) and sequenced by the BigDye Terminator Cycle Sequencing Ready Reaction Kit (Perkin-Elmer) on an automated DNA sequencer (ABI PRISM 3100 Genetic Analyzer, Perkin-Elmer) as previously described.^21^^,^^22^ The following reference sequences were used: *MYOC*: NC_000001.11, NM_000261.2, NP_000252.1, *OPTN*: NC_000010.11, NM_001008212.2, NP_001008213.1 (genomic, coding DNA, protein reference sequence, respectively).

### Determination of pathogenic variants based on the ACMG guidelines

Based on the American College of Medical Genetics and Genomics (ACMG) guidelines,^23^ pathologic variants were determined among variants identified by the comprehensive *TBK1* DNA CNVs, *MYOC*, and *OPTN* gene variants screening. In this study, we adopted the following criteria for determining pathological variants: (1) absence in the human population database and location in a mutational hot spot, (2) segregation of the variant from the family members, (3) proof of functional alteration of the protein by computational approach, and (4) evidence of dysfunction of the protein by well-established in vitro assay.

### Minor allele frequency filtering of *MYOC* and *OPTN* variants with a Japanese-specific reference panel

To extract pathogenic variants from the *MYOC* and *OPTN* variants of the POAG patients identified in this study, we examined the minor allele frequency (MAF) for each variant using a reference panel constructed by genome sequencing of a healthy Japanese population of 8380 individuals recruited as part of a prospective cohort study by Tadaka, S. et al (https://jmorp.megabank.tohoku.ac.jp).^24^ For the *OPTN* p.(Asn51Thr) variant, which was absent in the database and adjacent to the *OPTN* p.(Glu50Lys) variant, a well-characterized pathogenic variant of NTG, we implemented further segregation and functional analyses of the protein.

### Segregation of *OPTN* p.(Asn51Thr) variant in Japanese NTG families

Next, we performed segregation analysis from multiple affected family members of NTG patients with *OPTN* p.(Asn51Thr) variants. Genomic DNA was collected from the probands’ mother and sister with a confirmed diagnosis of glaucoma. The *OPTN* p.(Asn51Thr) variant was confirmed using Sanger sequencing as described above. To validate the *OPTN* p.(Asn51Thr) variant, a case with confirmed *OPTN* p.(Glu50Lys) variant was used as a positive control.^25^ Furthermore, ophthalmologic clinical data, including fundus photographs, optical coherence tomography images, and visual field examinations, which were available from the patients were obtained.

### In silico assessments of the effect of *OPTN* p.(Asn51Thr) protein on the binding stability of OPTN-TBK1 complex

To investigate the protein dysfunction caused by the *OPTN* p.(Asn51Thr) variant, we first used DynaMut (https://biosig.lab.uq.edu.au/dynamut/)^26^ to predict the protein stability changes upon the variant. Briefly, based on the crystal structure of the complex of the N-terminals of wild-type OPTN and the C-terminals of TBK1 (PDB https://doi.org/10.2210/pdb5EOF/pdb), we compared the stability changes of the OPTN-TBK1 complex upon *OPTN* p.(Asn51Thr) variant with those of *OPTN* p.(Glu50Lys) and wild-type *OPTN*. Moreover, we mathematically simulated the binding stability of the OPTN-TBK1 complex using PyMol (https://pymol.org/2/) and GROMACS 2020.4 (https://doi.org/10.5281/zenodo.3562512). In this simulation, 30PRO - 101SER and 679SER - 719MET were used for OPTN and TBK1, respectively, and long-range capacitance was calculated by the Particle-Mesh-Ewald method. The production run was performed for 100 ns in 2 fs steps. We also predicted the alterations of protein stability in a newly identified *MYOC* p.(Thr293Met) variant near the olfactomedin domain, for which the wild-type crystal structure (PDB DOI: https://doi.org/10.2210/pdb4WXQ/pdb) was available.

### In vitro protein solubility assays for *OPTN* p.(Asn51Thr) protein

To further confirm the protein functional changes caused by the *OPTN* p.(Asn51Thr) variant, protein insolubility in *OPTN* p.(Asn51Thr) variant was examined in vitro. pEF-Bos-FLAG-N51T (Asn51Thr) plasmid was obtained using PrimeSTAR Mutagenesis Basal Kit (TaKaRa) with pEF-Bos-FLAG-Optineurin as a template. Protein solubility assays were carried out as previously described.^19^ Briefly, HEK293T cells were transfected with pEF-Bos-FLAG-Optineurin, pEF-Bos-FLAG-E50K(Glu50Lys), or pEF-Bos-FLAG-N51T(Asn51Thr) plasmid and extracted by 24 hours after transfection. Soluble and insoluble fractions were analyzed by western blotting using anti-FLAG M2 monoclonal antibody (F1804, 1/10,000; Sigma-Aldrich) and anti-GAPDH (sc-32233, 1/2000; Santa Cruz Biotechnology).

## Results

### Screening for DNA CNVs in *TBK1*

We initially screened glaucoma patients for *TBK1* DNA CNVs. In the TaqMan copy-number assay, the previously identified case of *TBK1* duplication showed a copy-number value of 2. In contrast, the control group showed a value of 1, suggesting that the experimental protocol is highly reliable. Under the same conditions, none of the glaucoma patients in the study reached a *TBK1* copy number of 2 (Supplemental Figure 1). To eliminate the possibility of *TBK1* duplications or triplications, we subsequently performed a highly sensitive CNV absolute quantification using ddPCR. As a result, the possibility of *TBK1* duplications or triplications in these samples was ruled out. Collectively, we concluded that the glaucoma patients in this study do not have *TBK1* duplications or triplications.

### Determination of *MYOC* rare variants

Next, we used Sanger sequencing to determine the nucleotide sequence of the coding region of *MYOC* in glaucoma patients. Rare variants in the protein-coding regions of *MYOC* were then extracted using the Japanese-specific genome database. First, the *MYOC* variant encoding p.(Gln368Ter) in the olfactomedin domain, frequently found in European ancestry,^27^ was not found in the glaucoma samples in this study. In contrast, 4 rare *MYOC* variants with MAF < 0.01 were identified in the signal peptide and globular domain, of which *MYOC* p.(Cys5Trp) and p.(Thr293Met) were variants not present in the database (Table 1, Figure 2A, and Supplemental Table 3). Of note, both POAG patients with *MYOC* p.(Cys5Trp) or p.(Thr293Met) variants had severe glaucomatous optic neuropathy (Supplemental Figures 2 and 3), in which p.(Thr293Met) showed protein instability (ΔΔG: −0.120 kcal/mol, Supplemental Figure 3). Intriguingly, *MYOC* p.(Cys5Trp), p.(Gly12Arg), and p.(Thr256Met) variants were identified in patients with NTG (Table 1).

**Table 1:** POAG-related rare variants within *MYOC* and *OPTN* identified in this study

Gene	Genomic Change	Transcript Change	Protein Change	MAF (8.3KJPN)	Phenotype	ACMG Class
*MYOC*	NC_000001.11:g.171652597A>C	NM_000261.2:c.15T>G	NP_000252.1:p.(Cys5Trp)	**-**	HTG 1, NTG 1	Uncertain significance
*MYOC*	NC_000001.11:g.171652578C>G	NM_000261.2:c.34G>C	NP_000252.1:p.(Gly12Arg)	0.00251	NTG 1	Likely benign
*MYOC*	NC_000001.11:g.171636673G>A	NM_000261.2:c.767C>T	NP_000252.1:p.(Thr256Met)	0.0003	NTG 1	Uncertain significance
*MYOC*	NC_000001.11:g.171636562G>A	NM_000261.2:c.878C>T	NP_000252.1:p.(Thr293Met)	**-**	HTG 1	Likely benign
*OPTN*	NC_000010.11:g.13109274A>C	NM_001008212.2:c.152A>C	NP_001008213.1:p.(Asn51Thr)	**-**	NTG 1	Likely pathogenic
*OPTN*	NC_000010.11:g.13112486G>A	NM_001008212.2:c.403G>A	NP_001008213.1:p.(Glu135Lys)	0.00006	Unknown 1	Uncertain significance
*OPTN*	NC_000010.11:g.13112509G>C	NM_001008212.2:c.426G>C	NP_001008213.1:p.(Gln142His)	**-**	Unknown 1	Uncertain significance

Sanger sequencing was used to sequence the coding region of *MYOC* (NC_000001.11) and *OPTN* (NC_000010.11).

*ACMG* class, The American College of Medical Genetics and Genomics (ACMG) classification of variants; *HTG*, high-tension glaucoma; *MAF (8.3KJPN)*, minor allele frequency data of the Japanese genome reference panel from 8380 individuals; *NTG*, normal-tension glaucoma; *OAG*, open-angle glaucoma; *POAG*, primary open-angle glaucoma.

- Indicates variants not present in the database.

**Figure 2:**
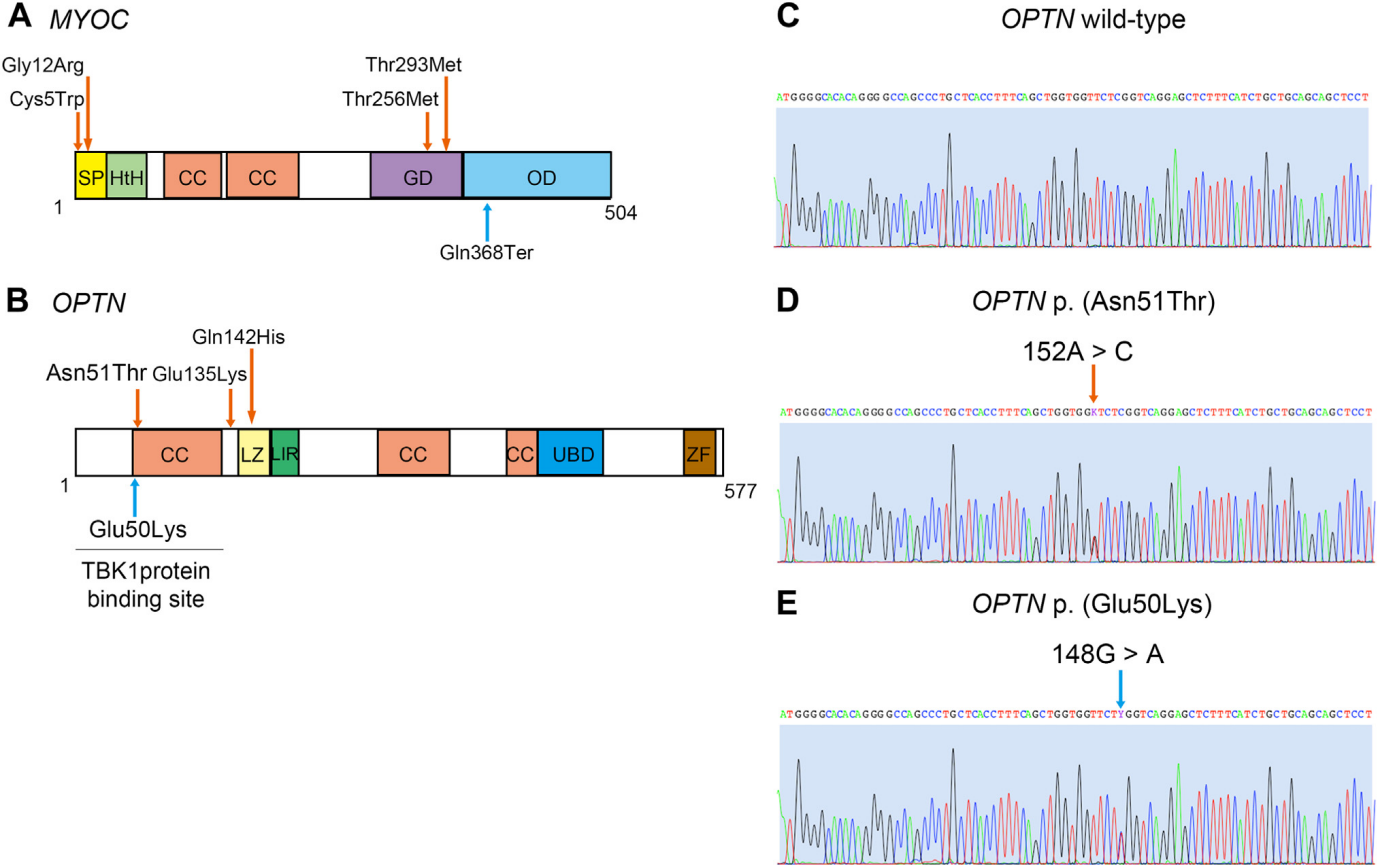
**Identification of novel POAG-associated rare variants within *MYOC* and *OPTN*.** A. A domain structure of the *MYOC* gene with 4 rare variants identified in this study: p.(Cys5Trp), p.(Gly12Arg), p.(Thr256Met), and p.(Thr293Met), as indicated in orange arrows. *MYOC* p.(Gln368Ter), the most common variant in European populations, as shown in a blue arrow, was not found in the POAG patients. CC, coiled coil; GD, globular domain; HtH, helix-turn-helix; OD, olfactomedin domain; SP, signal peptide. B. A domain structure of the *OPTN* gene with 3 rare variants identified in this study: p.(Asn51Thr), p.(Glu135Lys), and p.(Gln142His), as indicated in the orange arrows. *OPTN* p.(Glu50Lys), a well-known pathogenic variant in normal-tension glaucoma, was shown in the blue arrow. CC, coiled coil; LIR, LC3 interacting region; LZ, leucine zipper; UBD, ubiquitin-binding domain; ZF, zinc finger. C. Sanger sequencing chromatogram of the *OPTN* wild type. D. The orange arrow highlights a single base substitution of the *OPTN* p.(Asn51Thr) missense variant site in Sanger sequencing. E. The blue arrow highlights a single base substitution of the *OPTN* p.(Glu50Lys) missense variant site in Sanger sequencing.

### Identification of *OPTN* rare variants, notably p.(Asn51Thr)

After sequencing the coding region of *OPTN* in glaucoma patients in this study by Sanger sequencing, we used the reference panel to detect *OPTN* rare variants. No patients carrying the *OPTN* p.(Glu50Lys), a well-known pathogenic variant in NTG,^17^ were found. In contrast, 3 new rare *OPTN* variants with MAF < 0.01 were identified close to the coiled-coil or leucine zipper, of which *OPTN* p.(Asn51Thr) and p.(Gln142His) variants were absent in the database (Table 1, Figure 2B, and Supplemental Table 3). Notably, *OPTN* p.(Asn51Thr), an amino acid missense variant adjacent to the *OPTN* p.(Glu50Lys) variant not present in the reference panel database, was identified from 1 patient with NTG (Table 1, Figure 2C, D, and E). Additionally, 1 POAG patient had 2 rare variants, *OPTN* p.(Glu135Lys) and p.(Gln142His), and presented with severe glaucomatous optic neuropathy (Supplemental Figure 4).

### Segregation analysis of pedigree with *OPTN* p.(Asn51Thr) variant

Next, we performed a segregation analysis from the proband's family members with the *OPTN* p.(Asn51Thr) variant. Sanger sequencing of *OPTN* using available DNA samples from a mother and sister diagnosed with NTG identified the *OPTN* p.(Asn51Thr) variant, as did the proband, suggesting an etiology for the *OPTN* p.(Asn51Thr) variant (Figure 3). Furthermore, ophthalmologic examinations revealed that the proband and mother with the *OPTN* p.(Asn51Thr) variant were associated with severe glaucomatous visual field defects (Figure 3).

**Figure 3:**
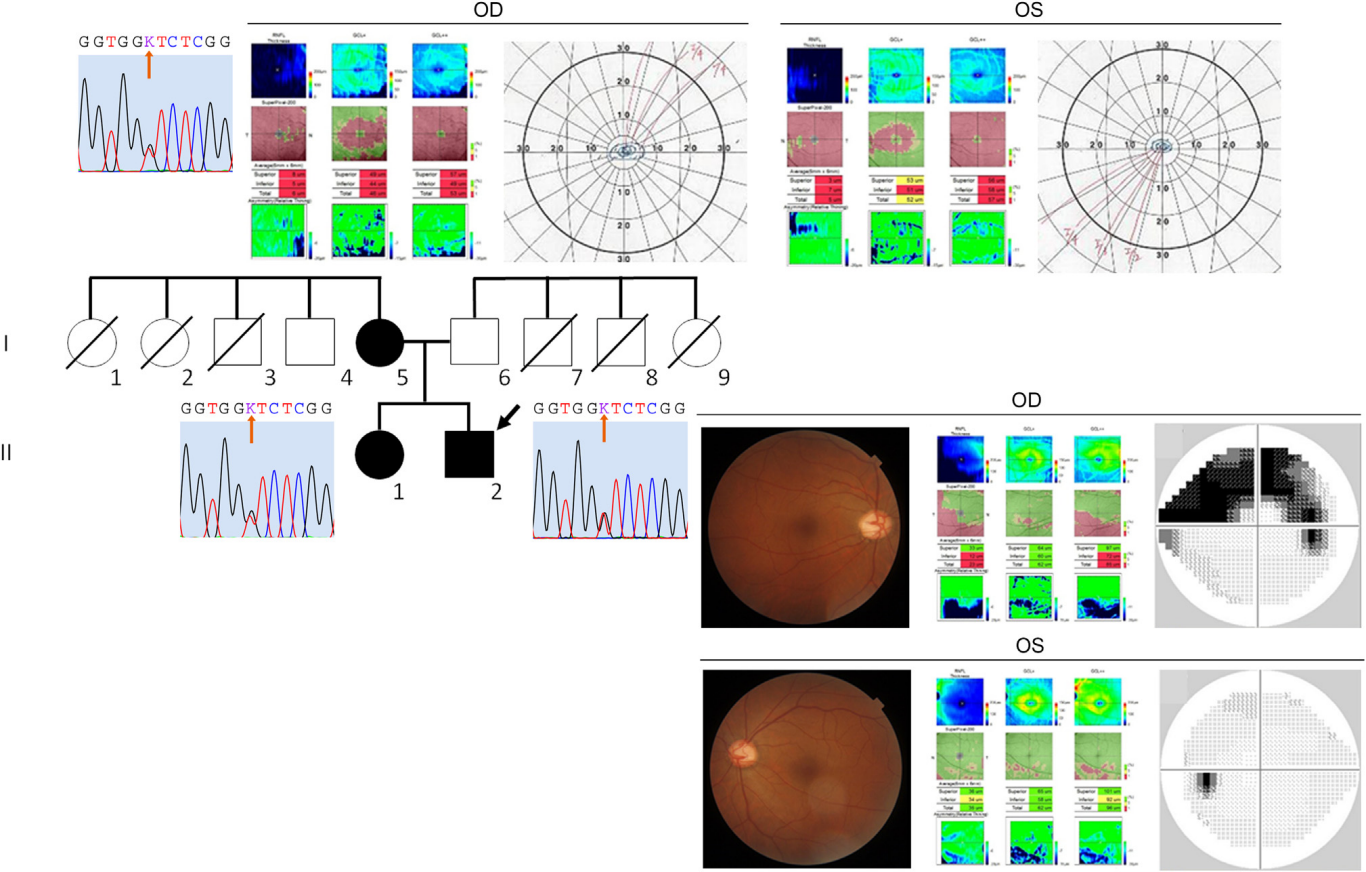
**Segregation analysis of *OPTN* p.(Asn51Thr) from an NTG pedigree.** Family members with normal-tension glaucoma (NTG) are indicated with black symbols, whereas those who did not meet the diagnostic criteria for glaucoma are shown as white symbols. Deceased members are displayed with slushes. A black arrow highlights a proband. Upper panels demonstrate a Sanger sequencing chromatogram of the *OPTN* p.(Asn51Thr) variant and ophthalmological examinations, including macular optical coherence tomography (OCT) scans and visual field data obtained by Goldmann perimetry in both eyes, from the proband’s mother (I-5). The left lower panel shows the *OPTN* p.(Asn51Thr) variant from the proband’s sister (II-1). The right lower panel depicts a Sanger sequencing chromatogram of the *OPTN* p.(Asn51Thr) variant and ophthalmological examinations, including fundus photos, macular OCT scans, and visual field examinations measured by Humphrey automated perimetry in both eyes, from the proband (II-2). OD, right eye; OS, left eye.

### In silico protein stability analysis of *OPTN* p.(Asn51Thr) protein

**Figure 4:**
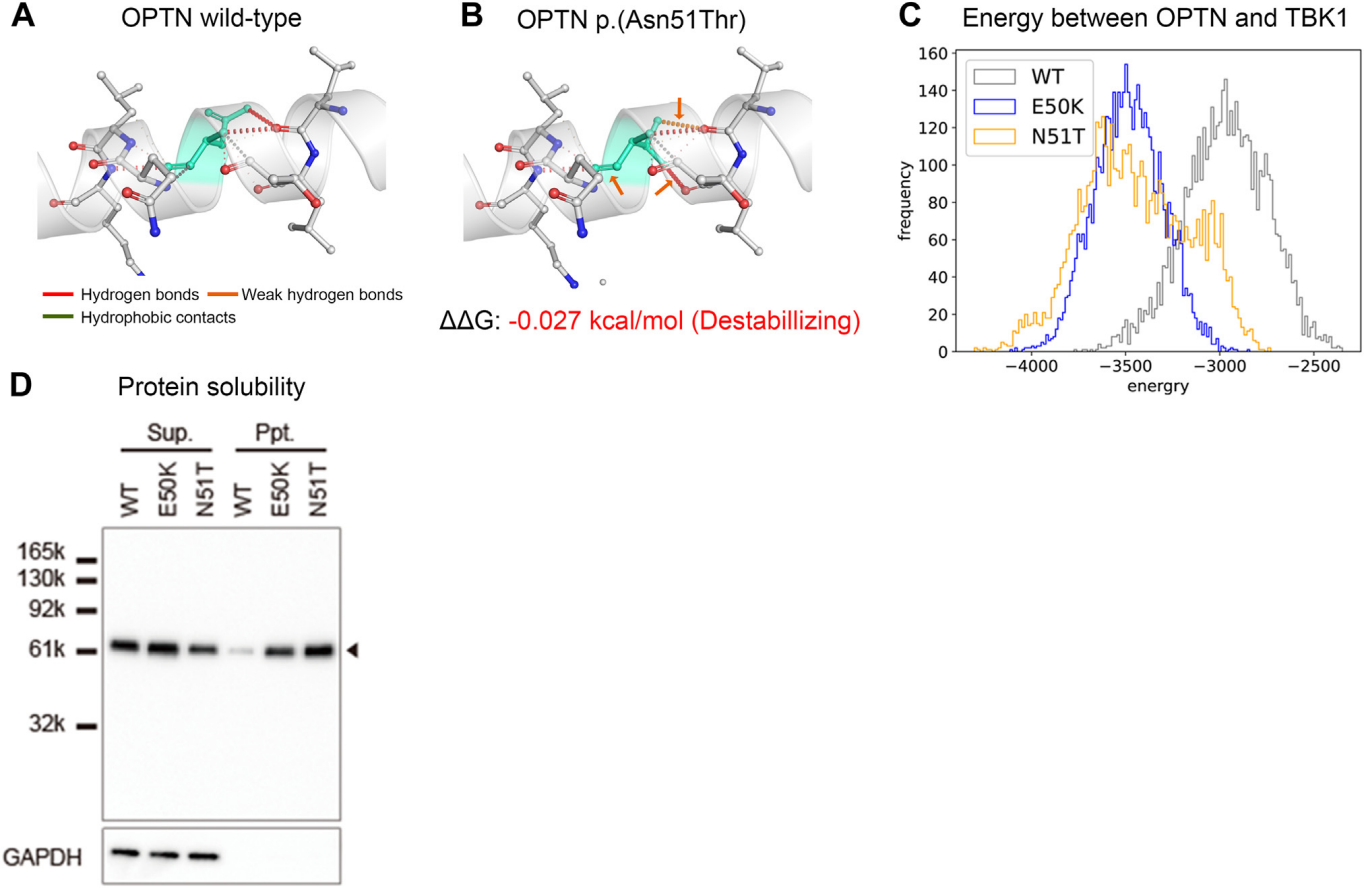
**Functional analysis of *OPTN* p.(Asn51Thr) protein.** A and B. *OPTN* wild type and p.(Asn51Thr) residues are colored in light green and are also represented as sticks alongside with the surrounding residues that are involved on any type of interactions. C. All-atom MD simulations for the *OPTN* wild-type, p.(Glu50Lys), and p.(Asn51Thr) and TBK1 complexes interaction energy showed that the conformation in *OPTN* p.(Asn51Thr) (N51T) is similar to that of *OPTN* p.(Glu50Lys) (E50K) than in *OPTN* wild type. D. In vitro analysis of *OPTN* p.(Asn51Thr) protein. Representative western blot image showing increased protein insolubility of the *OPTN* p.(Asn51Thr) variant (N51T), as well as the p.(Glu50Lys) variant (E50K). Same transfection condition of FLAG-tagged wild-type (WT), p.(Glu50Lys) or p.(Asn51Thr) proteins were analyzed by western blot using anti-FLAG-antibody. The black arrowhead indicates FLAG-tag band. Ppt, precipitation fraction; Sup, supernatant fraction.

The *OPTN* p.(Glu50Lys)-containing region is known to affect the TBK1 protein binding site^28^ (Figure 2B). Therefore, we sought to investigate the effect of *OPTN* p.(Asn51Thr) on the binding stability of the OPTN-TBK1 complex using DynaMut. We found that the *OPTN* p.(Asn51Thr) protein has altered hydrogen bonds and hydrophobic contacts compared with the *OPTN* wild type, resulting in OPTN-TBK1 complex instability (ΔΔG: −0.027 kcal/mol, Figure 4A and B). Next, we performed all-atom MD simulations for the OPTN wild-type, p.(Glu50Lys), and p.(Asn51Thr) and TBK1 complexes and measured each chain-to-chain interaction energy. The resulting histograms of the inter-chain energies of the OPTN wild-type, p.(Glu50Lys), and p.(Asn51Thr) TBK1 complexes are shown in Figure 4C, with median and interquartile ranges of −2967.47 kJ/mol (3.12563), −3483.81 kJ/mol (2.54261), and −3444.83 kJ/mol (4. 01264), respectively. Thus, we confirmed that the conformation in *OPTN* p.(Asn51Thr) protein is similar to that of p.(Glu50Lys) protein than in *OPTN* wild type.

### In vitro protein solubility assays of *OPTN* p.(Asn51Thr) variant

It is known that protein insolubilization is enhanced in *OPTN* p.(Glu50Lys) variant compared with wild type.^19^ Therefore, we assumed that protein solubility is affected in *OPTN* p.(Asn51Thr) variant, as well as in p.(Glu50Lys) variant and performed in vitro assays. Western blot analysis of FLAG-tagged *OPTN* wild-type, p.(Glu50Lys), and p.(Asn51Thr) proteins in the precipitate fraction with anti-FLAG antibodies showed that *OPTN* p.(Asn51Thr) protein, similar to p.(Glu50Lys) protein, also showed increased protein insolubility (Figure 4D). Together with the computational analysis results in silico, we concluded that the *OPTN* p.(Asn51Thr) protein had characteristics similar to the p.(Glu50Lys) protein, including instability of the OPTN-TBK1 complex and changes in protein composition, such as an increase in the OPTN-insoluble fraction.

## Discussion

Through a comprehensive *TBK1* DNA CNVs, *MYOC*, and *OPTN* rare variants screening, we identified a total of 7 rare variants, including 4 in *MYOC* and 3 in *OPTN*, in 6 of 174 Japanese POAG patients (3.4%). Despite the small sample size, 4 of the variants were novel and not present in the Japanese-specific reference panel. Next, we focused on the *OPTN* p.(Asn51Thr) variant adjacent to *OPTN* p.(Glu50Lys), a known pathogenic variant of NTG, among the 4 novel variants identified in this study. Based on the ACMG guidelines, we successfully segregated variants from the proband’s affected families and analyzed the function of the *OPTN* p.(Asn51Thr) protein using in silico and in vitro assays to explore the pathogenicity of the *OPTN* p.(Asn51Thr) variant.

We initially confirmed the absence of *TBK1* duplications and triplications in our POAG patients using TaqMan copy-number assay followed by ddPCR. Because a recent study in Korea using similar methods reported that *TBK1* duplications were detected in only 1 of 418 NTG patients (0.2%),^29^ the observation in the absence of *TBK1* duplications and triplications could be explained by the small sample size of this study. To identify POAG-related *MYOC* rare variants, we then used Sanger sequencing. *MYOC* p.(Gln368Ter), the most common variant in European populations,^27^ was not detected in the Japanese POAG patients in this study. A systematic review examining the frequency of *MYOC* rare variants in glaucoma patients in different ethnic populations using gnomAD demonstrated that *MYOC* p.(Gln368Ter) is more common in Finland (0.33%) and other European populations (0.11%) but null in East Asian ancestries,^30^ underscoring the importance of ethnic differences in rare variants.

Four rare variants: *MYOC* p.(Cys5Trp), p.(Gly12Arg), p.(Thr256Met), and p.(Thr293Met) were identified within *MYOC* in the POAG patients, 2 of which, *MYOC* p.(Cys5Trp) and p.(Thr293Met), were novel and not present in the Japanese-specific reference panel. Surprisingly, 2 *MYOC* rare variants in the signal peptide: p.(Cys5Trp) and p.(Gly12Arg) were detected in the present study, although less than 10% of glaucoma-related *MYOC* variants have been identified at the N-terminals.^14^ Myocilin proteins that consist of signal peptides are synthesized, folded, and processed in cellular organelles, including the endoplasmic reticulum and Golgi apparatus, and then secreted out of the trabecular meshwork cells.^14^ Similar to the *MYOC* p.(Cys25Arg) variant, which has been reported as one of the glaucoma-causing variants in the signal peptide sequence,^31^ the *MYOC* p.(Cys5Trp) variant is associated with cysteine residues and may impair normal secretion of myocilin.

In contrast, unlike myocilin proteins that comprise signal peptides, olfactomedin domain-resident *MYOC* variants are known to accumulate in the endoplasmic reticulum rather than being secreted, which can cause a toxic gain of function as a result of the intracellular protein misfolding.^14^ Given the C-terminal globular region of myocilin contains homology to olfactomedins,^32^ the *MYOC* p.(Thr293Met) variant within globular domain identified in this study, similar to other p.(Cys245Tyr), p.(Pro370Leu), and p.(Tyr437His) variants,^33^ may promote the formation of secretion-incompetent intracellular aggregates, linked to the induction of endoplasmic reticulum stress, thus triggering elevated IOP in POAG. However, further molecular biological validation is needed to fully understand the mechanisms underlying the accumulation of these myocilin protein variants. In addition, it is noteworthy that the 3 *MYOC* rare variants: p.(Cys5Trp), p.(Gly12Arg), and p.(Thr256Met), except p.(Thr293Met), were also detected in the NTG eyes. These results support previous studies that *MYOC* variants are also seen in patients with NTG.^15^

Next, we performed direct sequencing of the *OPTN* coding region in the POAG patients and found 3 rare variants: *OPTN* p.(Asn51Thr), p.(Glu135Lys), and p.(Gln142His), 2 of which, *OPTN* p.(Asn51Thr) and p.(Gln142His), were novel and not in the database. Intriguingly, one POAG patient had 2 rare variants of *OPTN*, p.(Glu135Lys) and p.(Gln142His), simultaneously. To the best of our knowledge, this is the first case report of multiple *OPTN* rare variants associated with POAG. The accumulation of such rare variants may accelerate the risk of developing glaucoma and disease severity at younger ages.

Remarkably, we segregated the *OPTN* p.(Asn51Thr) variants adjacent to a well-known pathogenic *OPTN* p.(Glu50Lys) variant^17^ from 1 NTG patient and the proband’s family members diagnosed with glaucoma. These findings suggest that this variant causes NTG, which led us to further analyze the function of the protein. Earlier studies have shown that the *OPTN* p.(Glu50Lys) protein enhanced TBK1-OPTN binding and increased protein insolubility.^19^ OPTN-TBK1 complex interactions and the resulting protein insolubility caused by the *OPTN* p.(Glu50Lys) variant may inhibit normal autophagy,^34^^,^^35^ (Huang KC, Gomes C, Shiga Y, et al. Autophagy disruption reduces mTORC1 activation leading to retinal ganglion cell neurodegeneration associated with glaucoma. bioRxiv. 2023. https://doi.org/10.1101/2023.01.04.522687) impair NF-kB activity,^36^ and increase oxidative stress,^37^ accelerating the vulnerability of RGCs to damage and subsequent death. In key experiments using in silico and in vitro assays, we provided evidence that the *OPTN* p.(Asn51Thr) rare variant, similar to *OPTN* p.(Glu50Lys), enhanced its interaction with TBK1 protein and increased insoluble aggregation, typically associated with the conformational change of the protein, suggesting a pathogenetic role for the *OPTN* p.(Asn51Thr) variant. In the future, transgenic mice and RGCs differentiated from human pluripotent stem cells harboring the *OPTN* p.(Asn51Thr) variant could be used to further elucidate the molecular mechanisms of disease development and discover therapeutic targets for these patients.

Our study had several limitations. First, the sample size was small, but, despite this, we succeeded in identifying 7 rare variants by enrolling POAG patients with a strong genetic predisposition. In the future, increasing the sample size may allow the identification of additional POAG-associated rare variants. Second, peak IOP was unavailable for all POAG patients, although 92.5% of the data were obtained. Third, central corneal thickness, diurnal IOP fluctuations, and the number of IOP measurements may affect IOP readings, leading to misdiagnosis of NTG in some patients. Lastly, for the *MYOC* and *OPTN* sequence data, control data obtained by the Sanger sequencing were not available. However, based on the ACMG guidelines,^23^ we used a genome sequencing database from a Japanese population of 8380 individuals, which reflects the accurate MAF of identified POAG-associated rare variants.

In summary, through a comprehensive *TBK1* DNA CNVs, *MYOC*, and *OPTN* rare variants screening, we (1) discovered 4 novel POAG-associated rare variants, *MYOC* p.(Cys5Trp), *MYOC* p.(Thr293Met), *OPTN* p.(Asn51Thr), and *OPTN* p.(Gln142His), (2) segregated the *OPTN* p.(Asn51Thr) variants from an NTG pedigree, and (3) confirmed functional alterations of *OPTN* p.(Asn51Thr) protein, as well as *OPTN* p.(Glu50Lys) protein, a well-known causal NTG variant. Our findings offer novel insights into rare variants of POAG in East Asians and provide evidence that *OPTN* p.(Asn51Thr) is likely a pathogenic variant in the development of this disease.

## Group Information

The members of the Japan Glaucoma Society Omics Group (JGS-OG) are Masato Akiyama, Kyushu University; Kazuhide Kawase, Yasuma Eye Clinic; Koji M Nishiguchi, Nagoya University; Fumihiko Mabuchi and Kenji Kashiwagi, University of Yamanashi; Takeshi Iwata, National Hospital Organization Tokyo Medical Center; Makoto Aihara, University of Tokyo; and Yu Yokoyama and Toru Nakazawa, Tohoku University.

## Data Availability

Further information and requests for the data of this study are available from the corresponding authors, except for primary patient sequencing data because they are derived from patient samples with unique variants that are impossible to guarantee anonymity for. Our institutional guidelines do not allow sharing these raw genome sequencing data because this is not part of the patient consent procedure.

## ORCID

Yukihiro Shiga: http://orcid.org/0000-0001-5531-7409

## Conflict of Interest

The authors declare no conflicts of interest.

## Figures and Tables

**Figure 1:**
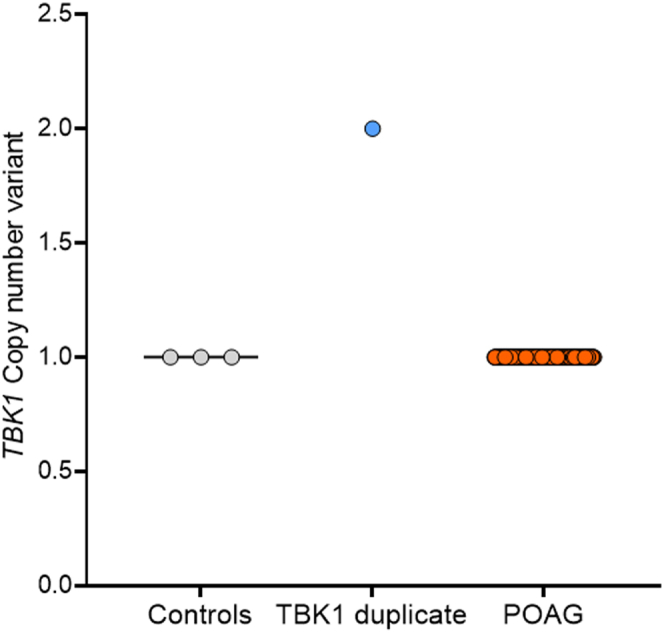


**Figure 2:**
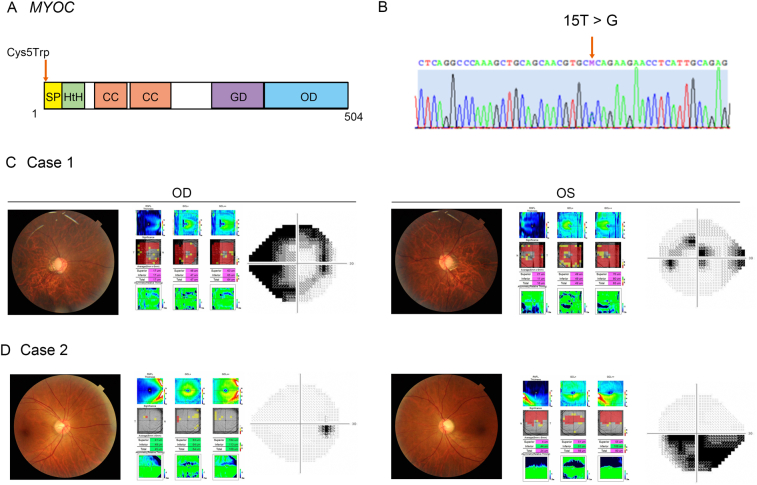


**Figure 3:**
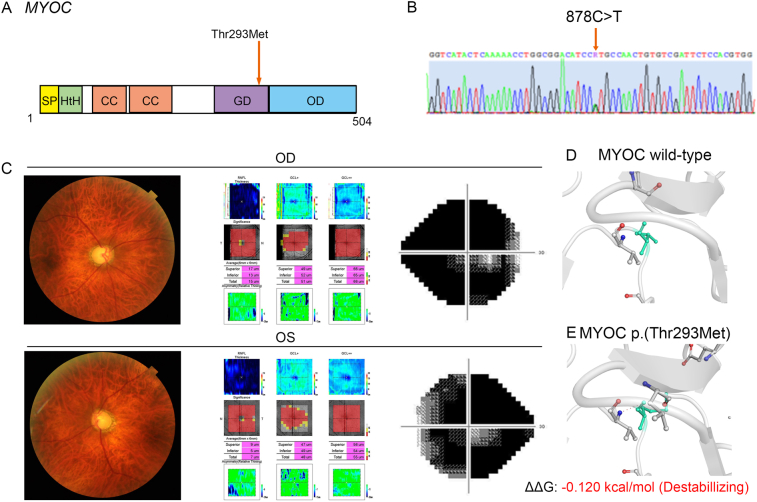


**Figure 4:**