# Supplementary material for: Identification of OPTN p.(Asn51Thr): A novel pathogenic variant in primary open-angle glaucoma
Source: Genet Med Open. 2023 Oct 31;2:100839. doi: 10.1016/j.gimo.2023.100839 (PMC11613796; doi:10.1016/j.gimo.2023.100839)
Supplement: Supplementary Table 1 [file mmc1.docx]

**Supplementary Table 1. Demographics and clinical characteristics of POAG patients in this study**

| POAG patients (N = 174) | |
| --- | --- |
| Age | 49 (45 - 64) |
| Sex (Female : Male) | 90 : 84 |
| Phenotype (HTG : NTG : Unkown) | 44 : 117 :13 |
| Family history (%) | 72.4 % |
| IOP (mmHg) | 17 (15 - 22) |
| Visual field damge (dB) | -14.93 (-21.14 - -11.02) |
| Surgical intervention (%) | 23.8 % |

Data are expressed as median ± interquartile range unless otherwise indicated.

POAG, primary open-angle glaucoma; N, number of subjects; HTG, high-tension glaucoma; NTG, normal-tension glaucoma; IOP, intraocular pressure.
