# Supplementary material for: Identification of OPTN p.(Asn51Thr): A novel pathogenic variant in primary open-angle glaucoma
Source: Genet Med Open. 2023 Oct 31;2:100839. doi: 10.1016/j.gimo.2023.100839 (PMC11613796; doi:10.1016/j.gimo.2023.100839)
Supplement: Supplementary Table 2 [file mmc2.docx]

**Supplementary Table 2. List of primers used to sequence *MYOC* and *OPTN* in this study**

| Gene | CDS | Primer | Primer sequence (5'->3') | Product size |
| --- | --- | --- | --- | --- |
| *MYOC* | 1a | Forward | GTGATCGCTGTGCTTTCCTTA | 495 |
|  |  | Reverse | CCAAAGCTCGACTCAGCTC |  |
|  | 1b | Forward | GCCTGGTCCAAGGTCAATTG | 437 |
|  |  | Reverse | GGCTCCCCAGTATATATAAACCTCT |  |
|  | 2 | Forward | CATCTCTCATCTTGCCCTGTG | 494 |
|  |  | Reverse | GCAGCCTATTTAAATGTCATCCTCA |  |
|  | 3a | Forward | GGTGACCATGTTCATCCTTCTG | 473 |
|  |  | Reverse | AAGGTGCCATTGTCCTCTCC |  |
|  | 3b | Forward | GTAGCCACCCCAAGAATACG | 500 |
|  |  | Reverse | TGTCACATCTACTGGCTCTGC |  |
| *OPTN* | 1 | Forward | TAACTGGAGAGAAAGTGGGCAA | 413 |
|  |  | Reverse | CACCTATGGAAACGGGGAGAA |  |
|  | 2 | Forward | AGAGGGAGATTTGGTTCATCAGA | 401 |
|  |  | Reverse | AGGCAAAACACCAATCCAGAC |  |
|  | 3 | Forward | GTCTTGCTATGTTGCCCAGG | 440 |
|  |  | Reverse | TCCTCAATCCTTGGCTTGTG |  |
|  | 4 | Forward | AAGCTTCCTTGGGTTGCATG | 291 |
|  |  | Reverse | AGTTACTTCCTCAGGTCACAAC |  |
|  | 5 | Forward | GAGTCTCCACACCTTCCCTAG | 496 |
|  |  | Reverse | CAGTGGTTGCACAATCCTGGA |  |
|  | 6 | Forward | CACTTGCCTTTTACCTCTGTGTG | 500 |
|  |  | Reverse | CAATTGACACAGAGCAGGACAA |  |
|  | 7 | Forward | GGATTGATTCACCAGCCAGTC | 404 |
|  |  | Reverse | TGCTCACACATTAACTGGAACA |  |
|  | 8 | Forward | GGGTTCAGAAATATGGCCAGG | 473 |
|  |  | Reverse | CTCTGAGAGGTCTGCTAGGA |  |
|  | 9 | Forward | CAGAAGGTTGGGAGGCAAGA | 333 |
|  |  | Reverse | ATCAGAAGTTACAAACCCTAGATGC |  |
|  | 10 | Forward | ACCTCAGCCTCTCAATTCTAGG | 465 |
|  |  | Reverse | CACTGAGCACTTTCCAAATGTTTC |  |
|  | 11 | Forward | TGCTATCGGAATGTACCTGGA | 416 |
|  |  | Reverse | GCGCGAACACAGCTATTC |  |
|  | 12 | Forward | TCCCCTACTTCTGTGGACTG | 339 |
|  |  | Reverse | TCGGTGGGTAATGGATGGAG |  |
|  | 13 | Forward | GAAGCAGGTATCACTTGGAGA | 459 |
|  |  | Reverse | CCGGCCTGTTTTCTTTCTTTTA |  |

CDS : Coding Sequence
